# Effectiveness of Magnesium Sulfate for Post-Cesarean Pain Control: A Double-blind Clinical Trial: Magnesium Sulfate for Post-cesarean Pain

**DOI:** 10.31661/gmj.vi.3741

**Published:** 2025-06-21

**Authors:** Satinik Darzi, Amirhosein Shahabi, Morteza Partovian, Sahereh Arabian, Fatemeh Paknazar, Fatemeh Khari

**Affiliations:** ^1^ Abnormal Uterine Bleeding Research Center, Semnan University of Medical Sciences, Semnan, Iran; ^2^ Semnan University of Medical Sciences, Semnan, Iran; ^3^ Anesthesia Group, Kowsar Hospital, Semnan University of Medical Sciences, Semnan, Iran; ^4^ Social Determinants of Health Research Center, Semnan University of Medical Sciences, Semnan, Iran

**Keywords:** Magnesium Sulfate, Cesarean Section, Pethidine, Pain Control

## Abstract

**Background:**

The use of magnesium sulfate as a part of analgesia has been used in recent years. The purpose of this research is to investigate the effectiveness of magnesium sulfate in controlling pain after elective repeat cesarean section under spinal anesthesia.

**Materials and Methods:**

In this double-blind study, 78 women who were candidates for cesarean section were randomly selected and divided into two groups of control and intervention of 39 people. Before spinal anesthesia, in the intervention group was injected 50 mg/kg of magnesium sulfate intravenously, and in the control group was injected with the same volume of normal saline. Pain intensity was assessed using the Visual Analogue Scale at 6, 12, 18 and 24 hours after the surgery operation and was recorded in a checklist along with vital signs and possible complications. The results were statistically analyzed using version 25 SPSS statistical software.

**Results:**

There was no significant difference between the intervention and control groups in the presence of complications during recovery time and up to 24 hours after surgery (P0.05). The pain score during the first 6 hours after surgery was equal to 8.56±1.51 in the intervention group and 8.21±1.15 in the control group. At 24 hours after surgery, this amount decreased to 4.23±1.08 in the intervention group and 4.49±0.79 in the control group. Although there was a difference in pain scores between the intervention and control groups up to 24 hours after surgery, this difference was not statistically significant (P0.05).

**Conclusion:**

This study showed that the administration or non-administration of magnesium sulfate has no effect on increasing the time of postpartum analgesia and reducing the need for pethidine.

## Introduction

Cesarean section is one of the most common and important surgical procedures in the world so that the prevalence rate of primary cesarean section in women in all ages, races, and medical conditions has been increasing rapidly and has increased tenfold during the recent 70-80 years [1][2][3]. Cesarean section is classified as a moderate to severe surgical procedure in terms of postoperative pain intensity [4]. The uncontrolled cesarean section pain leads to adverse effects for mothers and fetuses so that it causes increased cardiac output, number of respiratory rate, oxygen consumption rate, increased catecholamine levels, and consequently metabolic acidosis in the fetus [5][6]. Therefore, according to the high prevalence of cesarean section and the high percentage of pain in mothers after surgery, it is essential to provide appropriate analgesia to patients during and after surgery.

The cause of labor pain is not yet fully understood, but the most probable causes of this pain are included as follows: hypoxic contracted myometrium, compression of nerve ganglia in the cervix and lower uterus, collapse of contracted muscle bundles, stretching of the cervix during dilation, and also stretching of the peritoneum covering the cervix [7].

In general, 15% of the cervix comprises smooth muscle.

As a result, conditions that causes a decrease in calcium and an increase in the concentration of cyclic monophosphate (cGMP), is due to relax and soften of smooth muscle. Therefore, it is possible that by improving the condition of the cervix and softening it, the pain and discomfort caused by cervical stretching during labor can be reduced to some extent [8][9][10].

Magnesium sulfate is one of the drugs that reduce calcium entry into the cell, increase cAMP, absorbs water in the cervix, and causes to create edema in the cervix, which can reduce pain [11].

Magnesium is a physiological calcium channel blocker and a non-competitive antagonist of NMDA receptors and, theoretically, could play a role in part of the molecular process of sensitization and regulate postoperative pain by blocking NMDA receptors [12][13]. In fact, magnesium sulfate has an important analgesic effect on the spinal cord and acts as an antagonist of the NMDA receptor in the spinal cord and blocks neuronal transmission by reducing the release of acetylcholine [14]. In other words, magnesium sulfate increases the level of water channel proteins by activating protein kinase A and increasing the phosphorylation of cAMP protein components. This osmotic property and water absorption of the cervix can improve cervical edema and ultimately soften it [15].

According to the mentioned issues, the purpose of this research was to investigate the effectiveness of magnesium sulfate in pain control after cesarean section in mothers.

## Materials and Methods

**Table:** Table1. The Investigation of Weight, BMI and Creatinine Variables of the Studied Pregnant Mothers

**Block Number**	**Block**
I	AABB
II	BBAA
III	ABAB
IV	BABA
V	ABBA
VI	BAAB

**Figure-1:**
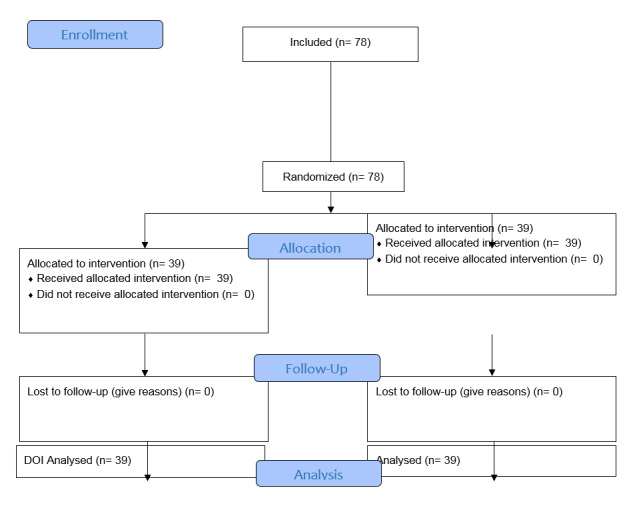
Figure 1. CONSORT Flow Diagram of the study

### Type of Research and Studied Population

This study is based on a double-blind clinical trial. The studied population includes all women candidates for elective repeat cesarean section under spinal anesthesia who referred to Amir al-Momenin Hospital in Semnan (Iran) between the years 2022 and 2023.

### Sampling Method and Sample Size

In this study, convenient (or available) sampling was used from the statistical population in order to refer the medical center and having the inclusion criteria between 2022 and 2023 and was selected to participate in the study using the randomized permuted blocks method into two equal groups of intervention and control. Based on this issue , and considering a significance level of 5% and a power of 80%, the final sample size of 78 people was calculated using G*Power software (version X.X; Heinrich Heine University, Dusseldorf; Germany) and divided into two groups of 39 people, the intervention group as A group and control group as B group (Figure-1).

Thus, six permuted blocks of fourfold were considered from different states A and B (Table-1). Then, based on the random number table (Numbers 0 to 9), each block was assigned to numbers 1 to 6 based on its number and successive extraction of numbers was carried out. The numbers 1 to 6 were considered as the blocks selection criteria and passed through numbers zero, 7, 8, and 9.

Based on the random numbers obtained from the table, the blocks were selected consecutively and each of the four patients was assigned to one of the two intervention or control groups based on their corresponding block.

### Inclusion and Exclusion Criteria

The lack of any specific problem or underlying disease, and a history of previous elective cesarean section, fenestrated surgical incision, and informed consent for performing the study, were among the inclusion criteria. The women in first pregnancy - multiple pregnancies - known sensitivity to magnesium sulfate and pethidine or other local anesthetics - underlying cardiac, hepatic, and renal diseases - contraindications of spinal anesthesia - chronic use of analgesics or narcotics - midline or non-fenestrated surgical incision - receiving any other analgesic other than a single dose of NSAID during recovery and pethidine when needed - any abnormal problem during the surgical procedure - patients receiving magnesium sulfate for other reasons, including preeclampsia, were excluded from the study.

### Data Collection Tool

In this study, the data were collected using a researcher-made checklist, which consisted of two sections of demographic information including patient age, reason for cesarean section, body mass index, and clinical information obtained from the effect of magnesium sulfate during recovery and 6, 12, 18, and 24 hours after the surgery and its complications caused by it. The validity and reliability coefficient of the aforementioned questionnaire were calculated based on Cronbach's alpha, 0.92, which indicates the appropriate validity of this questionnaire.

### Work Method

In this double-blind study, after the approval of the plan at Semnan University of Medical Sciences and Health Services and the approval of the Ethics Committee in Medical Research to the ethics code with No (IR.SEMUMS.REC.1402.125) , this trial was registered at IRCT (No IRCT20230923059492N1) , 78 pregnant women who had all the inclusion criteria were included in the study among all women candidates for elective repeat cesarean section referred to Amir al-Momenin Hospital (AS) in Semnan (Iran) between 2022 and 2023 and were divided into two groups of 39 people of control and intervention group. All participants provided written informed consent before enrollment in the study.

They were fully informed about the study objectives, procedures, potential risks, and benefits. They were also assured that their participation was voluntary, and they had the right to withdraw from the study at any time without any impact on their medical care. Confidentiality of participant data was strictly maintained.

This study was conducted in the form of a double-blind clinical trial. So that the person who prescribed magnesium sulfate and placebo to the patient was different from the person who collected the information, and also the patient himself was not aware of whether he received magnesium sulfate or placebo.

After obtaining informed consent from the patients and providing full explanations about performing the study, continuous monitoring including non-invasive blood pressure, heart rate control, electrocardiography, and pulse oximetry was performed. Ringer's solution infusion at a rate of 6 cc/kg was started in all patients 15 minutes before the surgery and in addition, in the intervention group, according to the standard, a dose of magnesium sulfate at a rate of 50 mg/kg [16] was injected, and in the control group, the same volume of magnesium sulfate and normal saline were injected.

Spinal anesthesia for all patients was performed by one person with 12.5 mg of Marcaine using a 25 gage spinal needle from the T3-T4 level and before the start of the surgical incision using the pfannensteil method. In the intervention group, the infusion of 50 mg/kg was started, and in the control group, the infusion of normal saline was started at the same volume and rate, and continued until half an hour after the end of the surgery. The pain intensity was assessed using the Visual Analogue Scale at 6, 12, 18 and 24 hours after surgery, and the VAS score was recorded in a checklist along with vital signs and probable complications such as nausea, vomiting, headache, palpitations, shivering, sleepiness, infection and respiratory depression if any incidence. During postoperative recovery, all patients received a dose of NSAID and were prescribed pethidine if needed. The time of administration of the first dose of pethidine and also its total dose within 24 hours after cesarean section were measured. If any other analgesic was administered within 24 hours, the subjects were excluded from the study. After discharge from recovery, the level of pain and the level of sedation, nausea and vomiting, headache, hypotension and respiratory depression were assessed at 6, 12, 18 and 24 hours after cesarean section and recorded in a researcher-made checklist.

### Data Analysis Method

The obtained data were statistically analyzed using 25 version SPSS statistical software (Statistical Package for the Social Sciences is developed by IBM. The manufacturer's name is **IBM Corp.** and the software is headquartered in **Armonk, New York, USA). The comparison of two groups was performed with t-test in the case of normality assumption and otherwise with Mann-Whitney U test at 95% confidence level (P values less than 0.05 were considered significant). The comparison of qualitative variables was performed with Pearson's chi-square test at the same 95% confidence level and if statistical assumptions related to this test were not met, Fisher's exact test was used. Following data collection, a post-hoc power analysis was conducted using G*Power software to assess whether the study had sufficient power to detect a clinically meaningful difference in pain scores between the intervention and control groups.

### Ethical Considerations

All possible complications were explained to the patients in simple and understandable language and the patients were able to withdraw from the plan at any time and for any reason. Participation in the study did not mean deprivation of treatment and did not involve any additional cost for the patients. All information will remain completely confidential by the researcher and the researcher is committed to keeping their information.

## Results

**Figure-2:**
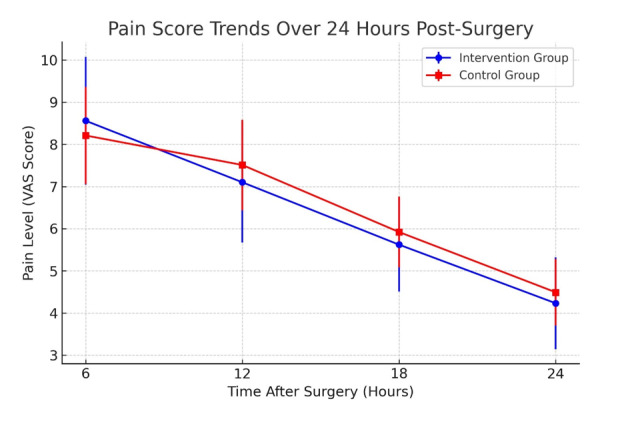
Figure 2. The pain based on VAS score within 6, 12, 18 and 24 hours after elective cesarean section in pregnant mothers

**Table:** Table2. The Investigation of Weight, BMI and Creatinine Variables of the Studied Pregnant Mothers

**Variable**		**Minimum**	**Maximum**	**Median**	**Interquartile Range**	**Mean**	**Standard Deviation**	**P-value**
**Age**	Intervention	17	42	30	11	29.67	6.2	0.999
	Control	20	39	29	8	29.67	4.99	
**BMI**	Intervention	24	45	29	5	29.82	4.29	0.755
	Control	22	40	30	6	29.92	4.21	
**Cr**	Intervention	0.4	0.9	0.6	0.2	0.61	0.11	0.344
	Control	0.5	0.9	0.6	0.2	0.43	0.11	

**Table:** Table3. The Information related to Taking Time of First Dose of Pethidine and the amount of Total Pethidine Received within 24 Hours After Elective Cesarean Section

**Variable**	**hours**	**Intervention**		**Control**		**P-value**
		**Number**	**Percentage**	**Number**	**Percentage**	
	3	8	20.5	4	10.3	
**Taking time of first dose of pethidine (hours) **	4	20	51.3	17	42.6	0.316
	5	9	23.1	16	41	
	6	2	5.1	2	5.1	
	25	0	0.0	4	10.3	
**Total pethidine received within 24 hours**	50	34	87.2	32	82.1	0.102
	75	5	12.8	3	7.7	

**Table:** Table4. The Information related to Possible Complications Attributable to Magnesium Sulfate within 6, 12, 18 and 24 Hours After Elective Cesarean Section

		**Intervention**		**Control**		**P-value**
		**Number**	**Percentage**	**Number**	**Percentage**	
	Not	36	92.3	36	92.3	
**Complications within 6 hours**	Nausea	1	2.6	2	5.1	0.717
	Headache	2	5.1	1	2.6	
	Not	39	100	37	94.9	
**Complications within 12 hours**	Nausea	0	0.0	0	0.0	0.152
	Headache	0	0.0	2	5.1	
	Not	39	100	39	100	
**Complications within 18 hours**	Nausea	0	0.0	0	0.0	0.255
	Headache	0	0	0	0.0	
	Not	0	0.0	0	0.0	
**Complications within 24 hours**	Nausea	2	5.1	1	2.6	0.556
	Headache	37	94.9	38	97.4	

**Table:** Table5. The Pain Assessment based on VAS Score within 6, 12, 18 and 24 Hours after Elective Cesarean Section in Pregnant Mothers

**Variable**		**Minimum**	**Maximum**	**Median**	**Interquartile Range**	**Mean**	**Standard Deviation**	**P-value**
**Pain level within 6 hours after surgery**	Intervention	3	10	9	2	8.56	1.518	0.078
	Control	5	10	8	2	8.21	1.151	
**Pain level within 12 hours after surgery**	Intervention	5	10	7	2	7.10	1.429	0.096
	Control	5	10	7	1	7.51	1.073	
**Pain level within 18 hours after surgery**	Intervention	4	8	5	1	5.62	1.115	0.077
	Control	4	8	6	1	5.92	0.839	
**Pain level within 24 hours after surgery**	Intervention	1	7	4	1	4.23	1.087	0.206
	Control	3	7	4	1	4.49	0.79	

In this study, 39 women candidates for elective repeat cesarean section under spinal anesthesia were examined in each intervention and control group. According to the statistical results, the two groups were homogeneous in terms of individual variables (age, body mass index, gravidity, plasma creatinine level) and there was observed no statistically significant difference. In the intervention group, the lowest age was 17 years and the highest age was 42 years. The average age of the individuals in the intervention group was calculated equal to 29.67 ± 6.20 and in the control group equal to 29.67 ± 4.99 years which there was observed no statistically significant difference(P=0.999). The lowest BMI in the intervention group was equal to 24 and in the control group was equal to 22, and the highest BMI in the intervention group was equal to 45 and in the control group was equal to 40. The mean BMI in the intervention group was calculated equal to 29.82 ± 4.29, which had not significantly different from the mean BMI (29.92 ± 4.21) in the control group (P=0.755). Also, the average blood creatinine level in the intervention group was equal to 0.61 ± 0.11 and in the control group was equal to 0.63 ± 0.11, and there was observed no statistically significant difference in terms of creatinine level in both groups (P=0.344, Table-2).

In the examination of information related to the taking time of the first dose of pethidine after elective cesarean section in pregnant mothers referred to the Amir al-Momenin (AS) hospital showed that 20 (51.3%) of mothers in the intervention group, compared to 17 (43.6%) of mothers in the control group, were requested painkillers after four hours of the first dose, and there was observed no statistically significant difference between the intervention and control groups (P=0.316). Also, in examining the amount of total pethidine received within 24 hours after elective cesarean section in the intervention group, 34 (87.2%) of mothers required 50 mg of pethidine and 5 (12.8%) of mothers required an additional dose of pethidine (75 mg). In the control group, 32 (82.1%) of mothers required 50 mg and 3 (7.7%) of mothers required 75 mg of pethidine, which was almost similar to the intervention group and there was observed no statistically significant difference between the two groups (P=0.102, Table-3). Statistical studies showed that the longest time has been took for the mother to pass gas or stool after cesarean section was in the control group and 16 hours after cesarean section, and in the intervention group it happened 12 hours after cesarean section, but both of these times were within normal range and there was observed no significant difference between the control and intervention groups (P=0.205) The results showed that there was no significant difference in the presence of complications during recovery time between the intervention and control groups (P=0.314).

However, the examination of complications 6 hours after elective cesarean section showed that one among 39 mothers in the intervention group and two among 39 mothers in the control group experienced nausea. Also, two other mothers from the intervention group and one mother from the control group experienced headache, but there was observed no significant difference between these two groups in terms of the presence of complications 6 hours after elective cesarean section (P=0.717).

However, during 12 hours after the surgery, only two people from the control group had headaches. At 18:00, there was observed no problems in any of the subjects in both of group, but at 24 hours after elective cesarean section, in two among 39 mothers in the intervention group and one among 39 mothers in the control group experienced nausea. Based on the obtained results of statistical analyses, there was observed no significant difference in terms of possible complications attributable to magnesium sulfate in the intervention group compared to the complications in the control group (Table-4).

The examination of average pain in the intervention group and its comparison with the control group, which was performed based on VAS score, showed that the average pain during the first 6 hours after surgery was 8.56 ± 1.51 in the intervention group and 8.21 ± 1.15 in the control group. After 12 hours of surgery, the average pain in the intervention group decreased to 7.10 ± 1.42 and 7.51 ± 1.07 in the control group. After 18 hours of surgery, the average pain in the intervention group was recorded to 5.62 ± 1.11 in the intervention group and 5.92 ± 0.83 in the control group, and this number decreased to 4.23 ± 1.08 in the intervention group and 4.49 ± 0.79 in the control group at 24 hours after surgery. The statistical analyses of average pain showed that there was a relatively high difference between the pain scores in the intervention and control groups at 6, 12, 18, and 24 hours after surgery, but this difference was not statistically significant. The results indicated that the statistical power to detect a significant difference between the two groups at different time points postoperatively was below 50%. This suggests that the study may have been underpowered to identify small differences in pain intensity (Table-5 and Figure-2).

## Discussion

Magnesium sulfate is an intracellular cation with various physiological functions such as enzyme activation, nerve signal conduction and protein synthesis, and regulation of vasomotor tonicity. Magnesium sulfate has been used in various clinical conditions including preeclampsia, tocolysis, arrhythmia, myocardial ischemia, bronchial asthma, and postoperative shivering [16][17]. In a study by Lee et al. (2009), it was showed that magnesium sulfate at two doses of 30 mg/kg and 45 mg/kg bolus, preoperatively, was effective in reducing post-cesarean section pain [18]. In another study by Rio et al., the effectiveness of 50 mg/kg bolus and subsequently 15 mg/kg magnesium sulfate in reducing VAS and analgesic consumption in gynecological surgical patients under general anesthesia has been recorded [19].

On the other hand, the studies have not confirmed the effectiveness of magnesium sulfate in reducing cesarean section pain [20][21]. Although the reasons for these discrepancies are not clearly understood, but they may lie in the study designs which has been examined in patients under epidural anesthesia and causes that the postoperative pain less felt. In our study, like as other studies, a dose of 50 mg/kg of magnesium sulfate was used to reduce pain. The dose of magnesium sulfate administered in our study was within the usual range used in the treatment of preterm labor and preeclampsia, which has been proven to be safe for both the mother and fetus [20].

The present study, which was performed with the aim of the investigation of the effect of magnesium sulfate in reducing pain and subsequently reducing pethidine consumption after elective cesarean section in pregnant women referred to Amir al-Momenin (AS) Hospital in Semnan, there was observed on significant differences in reducing pain and reducing pethidine consumption based on the use or non-use of magnesium sulfate, maternal age, body mass index, and plasma creatinine levels. In fact, it can be acknowledged that many of the factors studied, including magnesium sulfate, maternal weight, body mass index, different gestational ages, and plasma creatinine levels, have no significant relationship with pain level reduction within 24 hours after cesarean section and subsequently a reduction in the total pethidine dose. In a study which was conducted by Mireskandari et al. (2015) regarding the effect of magnesium sulfate on pain level reduction after cesarean section, it was shown that the age and body mass index had no significant effect on pain reduction within 24 hours [22]. Also, in the study by Shah et al. (2018), which were used magnesium infusion as an adjunctive analgesic after cesarean section, it was stated that age and body mass index had no significant relationship with pain reduction in magnesium users [23].

In our study, the age and BMI information of both intervention and control groups were similar and therefore it can be said that age and BMI did not affect the results obtained. Helmi et al. conducted a study in January 2015 on the prevention of pain after cesarean section by magnesium sulfate and its comparison with ketamine [24]. This study was conducted as a single-blind trial. In this study, 60 pregnant women who were scheduled to undergo cesarean section under anesthesia, were examined. The amount of need for fentanyl, moderate blood pressure, and heart rate were monitored during surgery procedure. The level of pain and sedation, and nausea and vomiting were also assessed at 2, 6, 12, and 24 hours after surgery. Helmi et al. [24] showed that there was no significant difference in the time the patient requested for the first dose of analgesic in the control group and the group that received magnesium sulfate. Also, the total amount of analgesic received within 24 hours after cesarean section was similar in both groups. The level of maternal pain during 24 hours after cesarean section based on VAS score had no significant difference in the control group and the group receiving magnesium sulfate. However, this level was higher than in the group receiving ketamine [24].

Our study also showed that the time of taking the first dose of pethidine in both intervention and control groups was almost similar each other, and the administration or non-administration of magnesium sulfate had no effect on increasing the time of analgesia after delivery. Also, the received pethidine total dose within 24 hours was almost similar each other in both intervention and control groups, and the administration or non-administration of magnesium sulfate had no effect on reducing the need for pethidine administration. These results are consistent with the results obtained by Helmi et al. But they are not consistent with the results obtained by Rezaei et al. [16][22][24]. Also, the level of pain based on the VAS score criterion at 6, 12, 18, and 24 hours after cesarean section showed that the p value at all 4 measured times is greater than 0.05, and as a result, it can be said that the administration of magnesium sulfate has no effect on reduce the amount of pain at 6, 12, 18, and 24 hours after pain. These results are consistent with the obtained results by Helmi et al., but they are contrary to the obtained results by Rezaei et al. [16][24] .

Also, in the examination of possible complications attributable to magnesium sulfate in mothers during recovery time, at 6, 12, 18, and 24 hours after cesarean section, complications such as cardiac and respiratory depression, sedation, and hypotension were not observed in any of the patients. The other complications such as nausea and headache were seen in both groups, but considering the p values greater than 0.05 in all cases, the prevalence of nausea and headache at different times during 24 hours after cesarean section was similar in both groups and they cannot be related to the administration or non-administration of magnesium sulfate. These results are consistent with the results of Rezaei et al., Yazdi et al. [16][25].

The existing literature on the effectiveness of magnesium sulfate for post-cesarean analgesia presents both supports and contradicts. Several factors may contribute to these discrepancies, including, differences in anesthetic techniques (spinal, epidural, or general anesthesia) and the use of adjunct anesthetics (e.g., bupivacaine, fentanyl) may influence pain perception and the analgesic effect of magnesium sulfate.

Furthermore, the dosage, timing, and route of administration vary significantly across studies, with some using a single bolus dose and others employing continuous infusion. Intravenous, intrathecal, and epidural routes may also result in different analgesic effects. Moreover, variations in patient demographics, including age, BMI, baseline pain thresholds, and comorbidities, may affect individual responses to magnesium sulfate and contribute to inconsistent findings. Some studies have administered NSAIDs or opioids alongside magnesium sulfate, which may have confounded the true analgesic effect of magnesium. Differences in multimodal analgesia protocols could explain the variability in reported outcomes.

In addition, differences in pain assessment scales (e.g., VAS vs. NRS), follow-up durations, and outcome measures may lead to variations in reported analgesic efficacy. Additionally, study design factors such as sample size and blinding methods could contribute to inconsistent results. These factors highlight the complexity of evaluating the efficacy of magnesium sulfate for post-cesarean analgesia and suggest that future studies should aim for standardized protocols to improve comparability across trials.

However, in this study, the limited sample size and the lack of cooperation or incorrect cooperation of some mothers were among the limitations of this research. Although the sample size was initially determined based on a priori power analysis to achieve 80% power for detecting a meaningful difference in pain scores, the post-hoc power analysis revealed that the actual power of the study was lower than anticipated. This discrepancy may be attributed to unexpected variability in the data distribution and lower-than-expected pain score differences between groups. Consequently, the study may have been limited in its ability to detect minor but potentially relevant differences in pain levels. Future studies should consider increasing the sample size or employing alternative statistical approaches, such as non-parametric tests, to enhance analytical precision. Furthermore, the study primarily focused on the short-term analgesic effects of magnesium sulfate, with pain relief assessed over a 24-hour post-surgery period. While this timeframe provides important insight into immediate pain control, a longer follow-up period could offer a more comprehensive understanding of the sustained effects of magnesium sulfate. This limitation should be considered when interpreting the findings.

## Conclusion

This study showed that the administration or non-administration of magnesium sulfate had no effect on increasing the duration of postpartum analgesia, and also the administration or non-administration of magnesium sulfate had no effect on reducing the need for pethidine.

## Conflict of Interest

The authors had no conflict of interest in conducting the present study.
